# An overview on amyotrophic lateral sclerosis and cadmium

**DOI:** 10.1007/s10072-020-04957-7

**Published:** 2020-12-05

**Authors:** Riccardo Oggiano, Andrea Pisano, Angela Sabalic, Cristiano Farace, Grazia Fenu, Simone Lintas, Giovanni Forte, Beatrice Bocca, Roberto Madeddu

**Affiliations:** 1 Department of Biomedical Science – Histology, University of Sassari, Sassari, Italy; 2 National Institute of Biostructures and Biosystems, Rome, Italy; 3 Department of Environment and Health, Istituto Superiore di Sanità, Rome, Italy

**Keywords:** Amyotrophic lateral sclerosis, Cadmium, Motor neuron disease, Heavy metals, Neurodegenerative disease

## Abstract

The present review represents an update about the knowledge of the possible role of Cadmium (Cd) in amyotrophic lateral sclerosis (ALS) initiation and its progression. ALS is a neurodegenerative disease that occurs in adulthood; its etiology is unknown and leads to death within a few years from its appearance. Among the various possible causes that can favor the development of the disease, heavy metals cannot be excluded. Cadmium is a heavy metal that does not play a biological role, but its neurotoxicity is well known. Numerous in vitro studies on cell and animal models confirm the toxicity of the metal on the nervous system, but these data are not accompanied by an epidemiological evidence, and, thus, an unclear correlation between Cd and the onset of the disease can be pointed out. On the other hand, a possible multifactorial and synergic mechanism in which Cd may have a role can explain the ALS onset. More efforts in new clinical, biochemical, and epidemiological studies are necessary to better elucidate the involvement of Cd in this lethal disease.

## Introduction

Amyotrophic lateral sclerosis (ALS) is a fatal neurodegenerative disease that occurs in adulthood. It is characterized by the progressive loss of motor neurons in the motor cortex, brain stem, and spinal cord. The disease causes muscle wasting and paralysis and usually leads to death within 2–5 years of its onset [1]. ALS patients can be classified into two groups: sporadic (ALSs) and familial (ALSf). ALSs represents about 90–95% of cases while ALSf the remaining 5–10% [2]. The average age at disease onset is ca. 60 years for the sporadic form and ca. 50 years for the family one [1]. It has a worldwide incidence of about 2 cases per 100,000 subjects and a prevalence that varies from 4 to 7 cases per 100,000 subjects [3], with uniform rates in Caucasian populations and lower rates in African, Asian, and Hispanic populations [4]. In most studies, ALS is more common in men than women [5, 6]. In European populations, the incidence of ALS has been estimated at 2.6–3.0 cases per 100,000 people [7]. Another paper reported the incidence of the disease in 10 countries of different geographical regions, and a higher prevalence was found in Uruguay, New Zealand, and the USA (age group was between 60 and 79 years) while a lower one in Serbia, China, and Taiwan. Besides, the same study estimated an increase in ALS cases worldwide of 69% from 2015 to 2040 [8]. A plethora of molecular and behavior-related mechanisms are implicated in the occurrence of ALS, but the causal event remains unknown. In particular, the main pathophysiological mechanisms that contribute to motor neuron degeneration in ALS are oxidative stress, mitochondrial dysfunction, axonal transport impairment, excitotoxicity, protein aggregation, endoplasmic reticulum stress, neuroinflammation, and abnormal RNA processing [9]. Furthermore, there is an epidemiological and clinical evidence that among the risk factors of the disease, there are viruses, cyanobacterial toxins, magnetic fields, several chemicals, and heavy metals [10]. Heavy metals are a particular chemical class of elements, without any biological role in the human body but with a harmful and toxic effect also at very low concentrations. Heavy metals are naturally present in the earth and human activity released them into the air, water, and food. Among them, cadmium (Cd) is a toxic element for the human body (the seven most dangerous substance for human health [11]), and the International Agency for Research on Cancer indicated Cd and Cd compounds carcinogenic for humans (group 1) [12]. Cadmium is a rare element in the crust with a concentration in the lithosphere of 0.08–0.1 ppm, and natural deposits of Cd are not present, but it is in combination with other materials to form minerals [13]. 9 Human exposure to Cd occurs by inhalation, ingestion, or through the skin, where it is poorly absorbed. In particular, inhalation is the main route of absorption of Cd or by occupational or by cigarette smoking exposures with the accumulation of Cd in the lung [14]. The amount absorbed through the intestine is accumulated in the body for a long time due to the almost absent mechanism of elimination from the body. The intestinal absorption is higher in people with zinc (Zn), iron (Fe), and calcium (Ca) deficiency, conditions in which Cd uses its divalent nature to compensate for their absence [15]. After being absorbed, it is accumulated mainly in the liver and kidney [16]. Cadmium toxicity is involved in a high number of pathologies, both by direct and non-direct effect, also in the nervous system with accumulation in the brain due to its ability to bypass the blood brain barrier [17, 18]. In human autopsies of individuals with diverse neurodegenerative diseases, it was observed an important accumulation of Cd in *locus ceruleus* [19]. Furthermore, other authors reported the direct correlation of the professional exposure to Cd and the development of the ALS in a worker of Ni-Cd battery factory [20]. Exposure to metal can be acute and chronic; acute exposure can cause pulmonary edema, while chronic exposure can cause kidney and bone damages [21]. Besides, the harmful effects of Cd in cells are worthy of consideration; in fact, apoptosis and necrosis effects [22], implication in oxidative stress by reactive oxygen species (ROS) generation [23], lipids peroxidation [24], and break of the mitochondrial membrane [25, 26] have been observed. Furthermore, Cd altered gene expression [27] induces unbalances in Ca homeostasis [28], altered the Zn and Cu metabolism [29], and has a role in various types of human cancers [30–32]. Exposure to Cd had a serious and diversified impact on the functionality of the nervous system with symptoms including headache and vertigo, olfactory dysfunction, Parkinsonian-like symptoms, slowing of vasomotor functioning, peripheral neuropathy, decreased equilibrium, decreased ability to concentrate, and learning disabilities [18]. Again, exposure to the metal is associated with a decrease of bone mineral density, resulting in osteoporosis and increased risk of fractures [33]. Cadmium could carry out its pathogenic effect in ALS, also taking part in a wider multifactorial mechanism, such as the altered balance of essential metals [34–36].

In this review, publications about the role of Cd in ALS were taken into consideration, especially in the latest 10 years (2011–2020). The publications were selected from three different databases (PUBMED, SCOPUS, and SCIENCE DIRECT), giving as keywords: “ALS Cd,” “Amyotrophic Lateral Sclerosis Cd,” “ALS Cadmium,” “Amyotrophic Lateral Sclerosis Cadmium,” and “Motor-Neuron Disease Cadmium.” The research had produced about 900 articles; after careful analysis, which included reading the abstracts of all the articles found, we selected and included in our work only those articles that seemed appropriate to our research, excluding book chapters and reviews. The number of the papers is 9 for the epidemiological part (two of these were made before 2011) and 11 for the laboratory one. In this order, we tried to update the state of the art to elucidate the knowledge about epidemiology and the latest developments, on the potential etiological mechanisms of Cd in this pathology.

## Epidemiological studies on cadmium exposure in ALS patients

Numerous studies have examined the effects of heavy metals on neurodegenerative diseases [37, 38]. Surveys have highlighted how the combination of geographical and epidemiological techniques was important for understanding the relationship between rare diseases and environmental exposure [39]. This fact linked with the unknown etiology of ALS leads researchers to speculate a relationship between the environment and its onset, including heavy metals. Exposure to heavy metals has been associated with the pathogenesis of ALS for over 150 years when heavy metals were detected in the tissues of patients with motor neuron diseases (MND) [40]. In Spain and France, three interesting studies have been carried out in the last 10 years to identify a relationship between exposure to environmental factors including heavy metals such as Cd and the onset of neurodegenerative diseases [39, 41, 42]. In particular, a French study analyzed the incidence of ALS within the region of Limousin, assessing the exposure of the population to environmental factors. It is interesting to note that municipalities with the highest number of ALS cases were more exposed to industrial activities; in fact, pulp and paper manufacturing industries have been significantly associated with a standardized incidence ratio (SIR) higher than 1 [41]. In 2018 in Spain, to identify the relationship between the occurrence of MND and exposure to heavy metals, the distribution of MND mortality rate in different municipalities over a period of 10 years was investigated by a spatial scale model [39]. The municipalities involved in the study were divided into exposed municipalities and unexposed municipalities, and results showed an increased risk of MND in exposed municipalities close to heavy metal emission sites. The study concluded that the combination of environmental emissions of different heavy metals including Cd (more than the single metal) was associated with a higher mortality rate (15.4%) in exposed sites than in control sites [39]. In the same year in Spain, a nested case-control study was conducted consisting of a retrospective cohort covering the entire region of Catalonia. The purpose of the study was to assess the long-term exposure of subjects to pesticides and air pollutants, including Cd, to understand whether the different geographical variations affected the occurrence of ALS. A linear association was found between Cd and the occurrence of ALS with an odds ratio (OR) = 1.332. Furthermore, an association was observed between Cd and benzopyrene, suggesting the possible impact of industrial emissions on the ALS occurrence [42]. In addition to studies based on geographical and epidemiological models, other authors have analyzed the concentration of Cd in different biological matrices of ALS patients and have investigated on how they differ from controls [43–46]. The human biological matrices mostly used were blood (intra-erythrocitary, exposure for a period of time equal to the life of a red cell), urine (short-time exposure), hair or nails (medium or a long time of exposure because of the affinity of Cd with keratin), and cerebrospinal fluid (CSF that may indicate accumulation in the brain). Bocca et al. [44] quantified Cd in blood, hair, and urine of subjects affected by ALS and controls. All the individuals enrolled in the study were non-smokers and non-drinkers and had never been exposed to heavy metals during their working life, and none of them had metal prostheses. The study was conducted in Sardinia (an island region of Italy) because of its high prevalence of ALS (ca. 8 cases per 100,000 subjects) in comparison with other regions in the world [47]. Results indicated that the content of Cd remained unchanged in blood, hair, and urine by comparing ALS patients and healthy controls. Besides, in the ALS group, females had more hair Cd than males. The results showed that Cd levels in blood were positively associated with Cd levels in the hair (*p* = 0.018, ρ = 0.400). However, the negative correlation between Cd content in hair and the duration of the disease did not prove the Cd-ALS relationship. The same ALS populations were sub-grouped accordingly to the severity of the disease. Results indicated that the patients with the more serious condition of the disease had blood Cd statistically higher than that in controls [46]. These two studies agree that the Cd is a neurotoxic metal [44], but that it is not directly involved in ALS; however, it can be a cause of concern if combined with other unleashed factors [46]. The research by Vinceti et al. [45] focused on the quantification of Cd in CSF of both ALS and control subjects, all living in the northern Italian region Emilia-Romagna. Concerning Cd results (exposure), the OR was decreased, while in the average tertile, it is higher, and the analysis for linear trend based on continuous values has confirmed an inverse association between exposure and risk [45]. Previously, the same authors analyzed the toenails of individuals affected by ALS and controls to measure concentrations of Cd [48]. This investigation, like the previous ones, did not suggest a key role of Cd in the etiology of pathology [48]. Again, concentrations of Cd in CSF and blood plasma were analyzed in patients from different regions of Norway [43]. In this case, the results showed that the CSF concentrations of Cd were higher in patients than in controls. This outcome seemed somehow to support an eventual involvement of Cd in ALS as reported also in previous studies. In 2001, Bar-Sela et al. [20] reported the case of a patient who died of ALS after 9 years of heavy professional exposure to Cd. The patient had elevated levels of urinary Cd roughly two orders of magnitude greater than levels seen in the general population. Cadmium blood levels, usually, fall rapidly following cessation of exposure, while Cd blood levels in this patient were high even after 6 months of exposure. His Cd blood levels were higher than those of his peers. The proteinuria, as found in this case, is correlated with protracting exposures to Cd [20]. Another study supporting the possible involvement of Cd in the pathogenesis of ALS was conducted in 2003 in the population of Guam [49]. In this study, the authors have determined the concentrations of Cd, Co, Cu, Fe, Mn, Rb, V, and Zn in formalin-fixed brain tissue. The concentrations of Cd were markedly and significantly elevated both in gray and white matter in ALS patients, while for other metals, no significant differences were detected [49]. As above reported, analysis of different human matrices is useful to have information about the Cd level in the body, but results were often contradictory when compared with other studies performed on similar matrices. This can be related to diverse reasons as different habits, working activity, geographical position, different analytical techniques, and, last but not the least, a very low Cd concentration in a non-exposed population. This may indicate that Cd alone could not be able to initiate the ALS, but it is more realistic to think that it is part of a more complex synergic mechanism (Table 1).

**Table 1:** Summary table of epidemiological studies conducted in the last 10 years, which relate the concentrations of Cd and other metals present within environmental, atmospheric and biological matrices

Year, authors [n° ref]	Type of study	ALS patients	Controls	Other metals	Matrices	Country
2011, Boumédiène et al. [41]	Observational	177	–	Pb, Hg, Al, Se, Mn, Cu, Fe	Environment	Limousin, France
2018, Sánchez-Díaz et al. [39]	Observational	9434 MND deaths in the period 2007–2016	–	As, Cr, Cu, Pb, Hg, Zn	Rivers	Spain
2018, Povedano et al. [42]	Observational: nested case-control	383	383	Pb, Hg, Se, Fe, Mn, Al	Environment	Catalonia, Spain
2015, Bocca et al. [44]	Observational: case-control	34	30	Al, Pb, Mn, Hg	Blood, urine, hair	Sardinia, Italy
2018, Oggiano et al. [46]	Observational: case-control	34	30	Al, Pb, Mn, Hg, Zn, Cu, Ca, Fe, Se, Mg	Blood, urine, hair	Sardinia, Italy
2017, Vinceti et al. [45]	Observational: case-control	38	38	Pb, Hg	CFS	Emilia-Romagna, Italy
2013, Roos et al. [43]	Observational: case-control	17	10	Al, Pb, Mn, Cu, Mo, V, U	CFS	Norway

## Cellular implication of cadmium in ALS models

On a cellular level Cd causes cell death, by different mechanisms, such as inducing apoptosis [50, 51], oxidative stress [52, 53], and by replacing essential metals at different levels for the cellular equilibrium by causing ions unbalancing [21] and by substituting them at levels of essentials proteins, such those involved in oxidative stress reduction, as Cu, Zn-SOD [54]. The etiological mechanisms of ALS are nowadays unclear, and the putative role of Cd in its insurgence and promotion is not effectively demonstrated, such as its putative mechanisms. In this section of the review, we report the last 10 years’ updates about the cellular mechanisms of Cd imputable to its role in ALS.

Oxidative stress represents a pathological condition in which the oxidant and antioxidant species lose their equilibrium in the cells, causing the loss of cellular functionality, by chemical alteration and inactivation of strategical enzymes that may cause cell death, as described in ALS and maybe at the base of the motor neuron death [21]. One of the mechanisms by which Cd causes oxidative stress is its capability to replace the Zn present in the superoxide dismutase (SOD) making it inactive. This alteration occurs in 10% of ALSf and 90% of ALSs. Huang et al. [21] observed the SOD concentration inhibition after Cd^2+^ addition at concentration > 500 nM, associated with a defective folding of the protein and Zn^2+^ decreased up to 1/7, without the effects on Cu^2+^ concentration. The results confirmed the ability of Cd^2+^ to replace the Zn^2+^, due to its higher affinity for the locus. In the same study, the treatment of N2A cell line with Cd^2+^ showed an increment of expressions of metallothionein (MT), thioredoxin, and peroxiredoxins 1 and 6, after Cd^2+^ treatment, justified by the authors as a protective mechanism against the oxidative stress induced by the high Cd^2+^ concentrations. The alteration of SOD and the consequent augmented expression of MT was observed also by Polykretis et al. [55], which purposed an alternative mechanism of SOD alteration by Cd^2+^, suggesting that Cd^2+^ induces Zn-SOD precipitation by the formation of an intermolecular disulfide bond [55]. Another way to induce oxidative stress in ALS is by induction of ROS, which naturally occurs in the cells, but an excessive production compromises cellular antioxidant defenses, with consequent free radical damage to cellular lipids, proteins, and nucleic acids. The ability of Cd^2+^ to induce ROS formation 36 times in respect to Na^2+^ and Mg^2+^ was demonstrated by Pogue et al. [56], in an in vitro model of ALS. To this end, Rahman et al. [54] studied the effect of Cd^2+^ on PC12 (neuron model), demonstrating its ability to induce ROS formation, and the increment of SH groups and LDH activity, phenomena reverted by the Zn^2+^ addiction. Otherwise, the excessive concentration of Zn^2+^ (500 μmol/L) showed a synergic toxic effect with Cd^2+^ [54]. Another mechanism by which Cd^2+^ induces cell damage is apoptosis. The induction of motor neuron apoptosis was demonstrated by the augmented VDAC1, a mitochondrial channel protein, involved in the induction of apoptosis, and the decreased of PDI, a protein, involved in the apoptosis outcome [21]. The above-cited study of Rahman et al. [54] showed that a concentration of Cd^2+^ at 5 μmol/L was sufficient to induce apoptosis and DNA fragmentation by activating endonucleases, and caspase 9, while a concentration of 10 μmol/L was necessaire to induce upregulation of cytochrome c, Bax, Bcl-2, and Bcl-X. The authors demonstrated the protective effect of Zn at a low concentration by restoring the Cd^2+^ induced alteration. Otherwise, when the Zn^2+^ was used at a concentration of 500 μmol/L, it shows a synergic effect with Cd^2+^ to induce cellular damage, by alteration of the mitochondrial function [54].

**Fig. 1:**
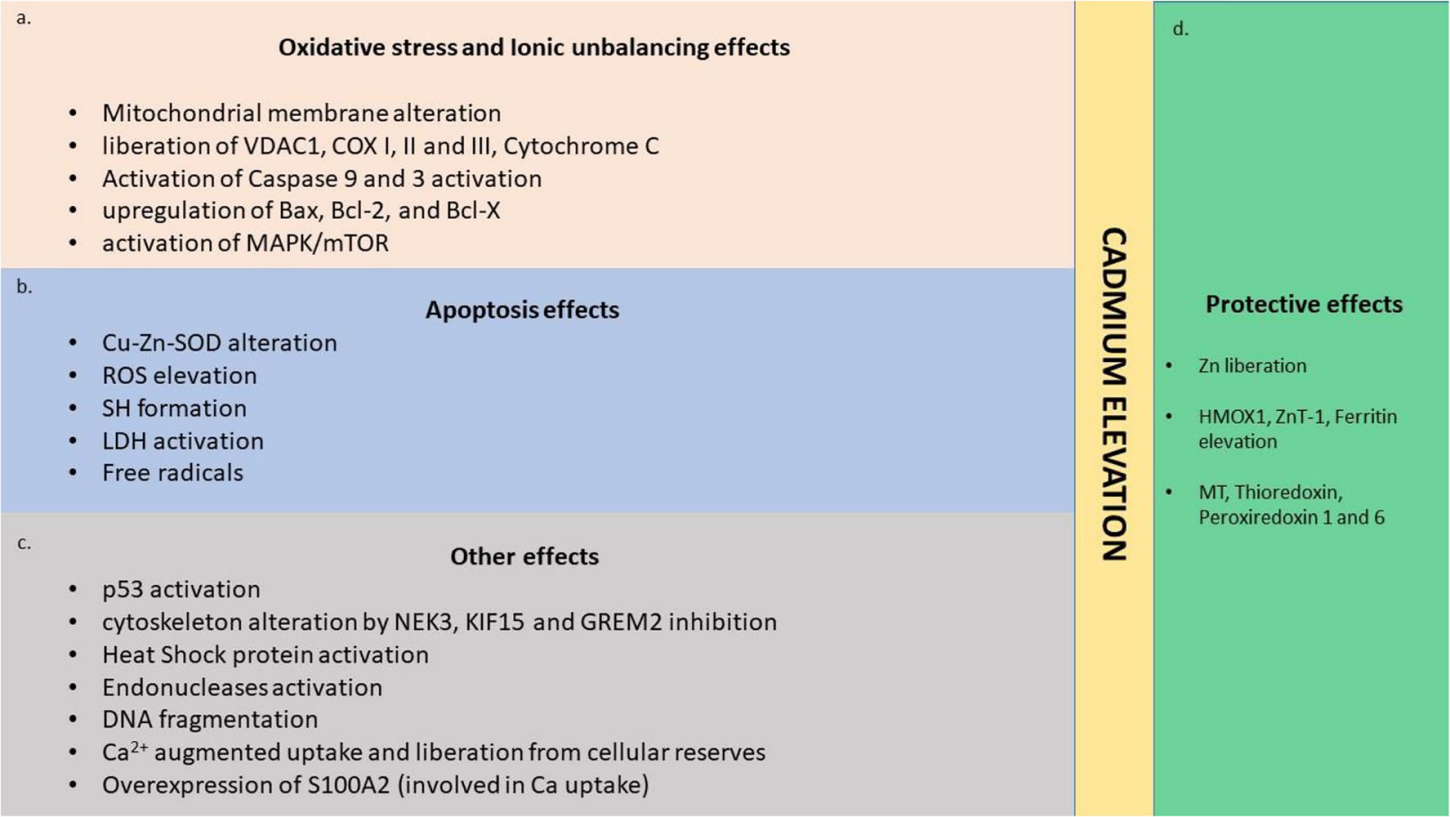
Graphical representation of the Cd’s effects on the motor-neuron. Each panel indicates the effect in a specific area of cellular damage: **a** oxidative stress and ionic unbalancing, **b** apoptosis, and **c** other effects; (**d**) shows the protective mechanisms induced in the cell to respond to the insult of cadmium

As seen before, Cd^2+^ is involved in the ionic unbalancing. In particular, the interaction between Cd^2+^ and Ca^2+^ has been widely studied, to understand if this could play a role in the damage of motor neurons and in the development and progression of ALS. In vitro and animal studies demonstrated that augmented concentration of Cd^2+^ induces elevation of Ca^2+^ concentration by its release from intracellular storage and by extracellular uptake [28]. The consequence of the intracellular increase of Ca^2+^ was cell death induction, with MAPK/mTOR activation, dysfunction of cytochrome oxidase subunits (COX-I/II/III), depletion of mitochondrial membrane potential, cleavage of caspase-9, caspase-3, and poly (ADP-ribose) polymerase (PARP); oxidative stress induction with ROS levels elevation [28, 57]; both the process were inhibited after Ca^2+^ chelation [28, 57]. Furthermore, it was observed that Cd^2+^ induces the overexpression of S100A2, a Ca^2+^-binding protein with an essential role that mediates signal transduction and diseases in the nervous system, such as multiple sclerosis, Parkinson, and ALS [58]. Taken together, these results indicate Ca^2+^ as a fundamental mediator of Cd^2+^ in its negative neuronal effect. The effect on glia, in particular on astrocytes, has been tested by Ospondpant et al. [59], demonstrating how intracellular Cd^2+^ increase, decrease the glutathione levels, increase Ca^2+^ content, altering mitochondrial membrane state end activate the JNK and PI3k/Akt pathways, to induce astrocyte apoptosis. The ability of Cd^2+^ to induce apoptosis and cell cycle arrest is analyzed in this study, and the authors observed that Cd^2+^ concentrations > 20 μM induce DNA fragmentation, Bcl2 downregulation, and Bax upregulation and the promotion of p53, p27, and p21 [59]. In conclusion, the idea of an involvement of Cd among the behavioral components implicated in the ALS insurgence seems to be supported by the literature of in vitro models, with different mechanisms such as alteration of antioxidants, cell cycle, and cell fate system, to induce motor neuron cell death (Fig. 1).

## Conclusion

Amyotrophic lateral sclerosis is a fatal neurodegenerative disease of unknown origin. Many hypotheses have been released of different origins as environmental and/or genetics, but none of them resulted conclusively. Among the possible factors, some heavy metals, such as Cd, are known to be neurotoxicant. In the last decades, studies were performed to verify if the Cd was able to promote the ALS. The data presented until now do not allow to attribute the direct involvement of Cd in ALS, but at the same time, it cannot be excluded. The role of Cd in ALS may be identified in a synergic multifactorial mechanism, in which it plays an important but probably replaceable role; other studies are necessary to better elucidate this eventual involvement in this pathology, such as studies of cellular assets in patients.
